# Self-Isolation and Quarantine during the UK’s First Wave of COVID-19. A Mixed-Methods Study of Non-Adherence

**DOI:** 10.3390/ijerph18137015

**Published:** 2021-06-30

**Authors:** Yolanda Eraso, Stephen Hills

**Affiliations:** 1 School of Social Professions, London Metropolitan University, London N7 8DB, UK; 2 Guildhall School of Business and Law, London Metropolitan University, London N7 8DB, UK; s.hills@londonmet.ac.uk

**Keywords:** COVID-19, self-isolation, quarantine, adherence behaviours, mixed-methods, UK

## Abstract

Self-isolation and quarantine measures were introduced by the UK Government on 12 March 2020 as part of the ‘delay’ phase to control the spread of SARS-CoV-2. Non-adherence to self-isolation for 7 days after the development of symptoms is considered suboptimal and little is known about adherence to quarantine for 14 days if a co-habitant developed symptoms. This study aims to analyse non-adherence behaviours to self-isolation and quarantine measures by identifying their potential psycho-social and demographic predictors and by exploring people’s accounts of their experiences with these measures. A mixed-methods convergent design was used, comprising an online survey (*n* = 681) completed by residents in six North London boroughs and qualitative interviews with a subsample of participants (*n* = 16). Findings identified not accessing community support, lack of control over leaving the house, and lack of perceived benefit and need to follow the rules as behaviours associated with non-adherence to quarantine (42.7%). Non-adherence to self-isolating measures (24.4%) was associated with individuals’ perceived lack of control over responsibilities, lack of control over leaving the house, uncertainty about symptoms experienced, lack of access to tests, and distrust in the Government. Adherence to self-isolation and quarantine could be improved through strengthening perceived benefit to self-isolate with messages emphasising its effectiveness, by implementing a two-way information system to support symptoms identification, and with Government-funded, locally supported packages at different levels (financial, food, and practical needs).

## 1. Introduction

Self-isolation (SI) is the separation of infected individuals and quarantine is the separation of healthy, asymptomatic individuals who have been in contact with suspected or positive cases. During the COVID-19 pandemic, countries across the world have drawn on these and other non-pharmacological public health measures to control the spread of the disease. Given the uncertainty surrounding the transmissibility of SARS-CoV-2, SI rules were soon extended to include not only confirmed cases, but symptomatic individuals (the case in the UK) or those whom health authorities suspected might be infected, even without symptoms, as set out in guidelines in Australia [1,2]. In the context of the containment policies to control the spread of COVID-19, the World Health Organisation (WHO) issued guidance [3] recommending 14 days quarantine (given the period of incubation) for those who were in contact with a confirmed case. In addition to consistent guidelines and communication, including engagement with communities to increase acceptability, and adequate spaces, WHO recommended that governments provide individuals in quarantine ‘with health care; financial, social and psychosocial support; and basic needs, including food, water, and other essentials. The needs of vulnerable populations should be prioritized’ [3] (p. 1). A Cochrane rapid review [4] found that quarantine was an effective measure for reducing the number of infections and mortality rate, and that it was most effective if started earlier and was implemented alongside other restrictive measures, such as social distancing and school closings.

In the UK, SI and quarantine measures were introduced in February 2020, with Public Health England (PHE) requiring individuals to self-isolate for 7 days if they have returned with symptoms from areas known to have COVID-19 cases, or without symptoms (quarantine) if the area was categorised as high risk [5]. Under the Government’s ‘contain phase’, PHE used testing of suspected cases at the main borders alongside contact tracing to inform, monitor and isolate those in close contact with positive cases [6]. The latter policy, however, was abandoned on 12 March 2020 [1], when the ‘delay’ phase was announced by the Government, requiring those with symptoms (a new, continuous cough and high temperature) to self-isolate at home for 7 days, and avoid all but essential contact with others. Individuals did not need to inform the National Health Service (NHS) and were advised to seek information remotely on the NHS 111 website, and to phone only if their symptoms did not disappear or became worse after 7 days [1]. In addition, quarantine measures were introduced for asymptomatic individuals within a household, and in contact with someone who developed symptoms, who needed to quarantine for 14 days since the day symptoms were identified in the co-habitant. On the 23 March 2020 [7], the ‘mitigation’ phase (first national lockdown) was implemented, with social distancing measures added to pre-existent self-isolation and quarantine measures.

SI and quarantine measures varied considerably across countries during the first months of the pandemic, where a conspicuous distinction emerged between Asia [South Korea, Taiwan, China and Singapore] and Europe, the US, Australia and New Zealand. Asian countries tended to introduce mass testing and contact tracing with digital surveillance alongside institutional isolation and enforcement, whilst Western countries were more inclined to home isolation measures, develop different levels of testing and contact tracing, and varied in their enforcement and penalties [8,9,10,11]. Countries such as Australia and New Zealand, for example, successfully introduced SI and quarantine measures, including strict control or closure of borders, and monitored quarantine [12] whilst many Western European countries restricted entries from EU borders, except the UK whose borders remined opened. Spain, Germany, and Norway introduced fines to enforce adherence with SI and quarantine, whereas in Sweden and the UK adherence was voluntary. In the latter, only on 28 September 2020 when the NHS Test and Trace was introduced, legislation incorporated fines for offenders [13]. SI and quarantine are difficult demands imposed on individuals. The separation and restriction of movement of symptomatic (SI) and asymptomatic individuals in contact with a suspected case (quarantine) is challenging, for which risks and benefits should be clearly communicated [3] and arguably, necessities and concerns of different population groups should also be acknowledged and supported. Similar to WHO guidance, a recent qualitative evidence synthesis [14] has found that strategies to increase adherence to quarantine should be effectively communicated by public health and other agencies involved (e.g., identification of symptoms, risk of transmission, specific actions to take in different scenarios), community oriented (e.g., working with local partnerships with groups to understand needs), provide financial compensation, food provision, social and psychological support, and mitigate against harms (e.g., stigma, social isolation, and vulnerable groups).

Whilst all the above factors can equally be applied to people required to self-isolate due to symptoms, these behaviours differ in terms of what is required for adherence: SI demands people’s self-recognition of symptoms that are bodily experienced by the individual, whilst quarantine of asymptomatic individuals demands a great deal of altruism (i.e., an understanding of the need to isolate for the benefit of the community), a belief that in the case of the UK needed to be sustained for a lengthy period of two-weeks. Such a distinction is evident in the differing rates of adherence to SI [15] and quarantine [16] in previous pandemics in Western countries, and also in COVID-19 research where a study in Norway [17] found that adherence to SI and quarantine differs depending on the absence or presence of symptoms: adherence was 71% when an individual had symptoms but dropped to 28% when symptoms were not present.

Some of the behaviours that have been identified as affecting adherence to quarantine measures in previous pandemics were knowledge about disease transmission and quarantine guidelines, social norms, self-efficacy to perform the behaviour, trust in government, support received, perceived susceptibility and severity of the disease [18]. Additionally, in the context of the COVID-19 pandemic, fatigue appears to be an issue whereby the Norwegian study [17] found that adherence to SI and quarantine peaked at 66% in April 2020 but dropped to 33–38% in May–July.

In the UK, there are no available data yet on the extent of quarantine adherence behaviour since the pandemic started, but for SI, a recent study [19] found that adherence has been low, with only 20.2% not leaving the house in the previous 7-days after developing the three most common COVID-19 symptoms (cough, high temperature, and loss of taste or smell). Only 51.5% participants identified these symptoms, thus knowledge was a limiting factor. The study also identified key demographic factors as associated with low adherence: living in a deprived area, lower socioeconomic status, having a dependent child, males, and younger people.

This paper contributes to the under-researched area of behaviours underpinning non-adherence to SI and quarantine measures, including an exploration of their similarities and differences to inform recommendations for the UK context. The study aimed to identify non-adherence behaviours to SI and quarantine measures, their potential predictors, and people’s accounts of their experiences. It follows a mixed-methods convergent design comprising an online survey and qualitative interviews in a sample of North-London residents. The following research questions guided this investigation: (1) What are the demographic, housing, health, political, psychological, and social factors associated with non-adherence of SI and quarantine rules after developing COVID-19 symptoms or after a co-habitant developed symptoms? (Quantitative phase); (2) What are participants experiences of SI and quarantine? (3) What do participants feel are the main reasons for non-adhering to the rules? What do participants belief would have made it easier for them to adhere to the rules? (Qualitative phase). The timeframe of the study spans the end of the first national lockdown and subsequent relaxation of measures (May–September 2020). Quantitative findings revealed that non-adherence of SI and quarantine rules were 24.4% and 42.7%, respectively, during a period when household infections in London, according to the Office for National Statistics (ONS), were low and slightly increasing from 0.1% in June to 0.2% in September [20]. The findings provide insight into individual, interpersonal and community level factors that have affected people’s adherence to SI and quarantine, and highlights the differences and similarities observed in these two distinct behaviours.

## 2. Materials and Methods

A mixed-methods convergent design consisting of an online survey and qualitative interviews with a subsample of participants [21] was undertaken. This design was selected to better understand the factors associated with non-adherence by addressing the known limitations that observational studies pose regarding causality, which are of relevance when attempting to interpret behaviours and inform interventions during pandemics [22]. In addition, qualitative studies, though inferentially rich, are usually restricted to smaller samples. Hence, the authors considered that research on non-adherence behaviours could be improved if the nature of the quantitative associations observed, including counter-intuitive findings, could also be explored with qualitative data reporting on the reasons for non-adherence in a subsample of participants.

In this design, data collection occurred at different periods, with each data analysed and presented separately, and integrated in Section 4. The process of data integration aimed to expand and complement different aspects of non-adherence of SI and quarantine behaviours. We used a ‘waving approach’ to integrate quantitative and qualitative results into key themes identified for each behaviour, SI and quarantine [23].

### 2.1. Quantitative

#### 2.1.1. Design

The quantitative component of the study was a cross-sectional survey administered via convenience sampling among adults in North London. Participants were required to be aged ≥ 18 years and a resident in the London boroughs (local authority districts) of Islington, Haringey, Camden, Hackney, Barnet, or Enfield. In order to achieve a 99% confidence level and 5% margin of error in relation to the 1,777,666 population of the qualifying boroughs [24], a minimum sample size of 663 was required [25]. A digital questionnaire using Joint Information Systems Committee (JISC)’s online surveys software was used, which meets information security standards and is General Data Protection Regulation compliant [26]. The questionnaire went live on 1st of May 2020 and closed on 31st of May 2020, when lockdown was eased for the second time. To encourage questionnaire completion an incentive of a random prize draw to win one of four £100 vouchers for the Aldi supermarket was used. A summary of the research and a link to the questionnaire were disseminated via the authors’ university website and social media accounts, local newspapers, and North London Facebook community groups.

#### 2.1.2. Instrument

A 15 min self-report questionnaire was developed, measuring SI and quarantine infringements and seven groups of factors hypothesised as being associated with non-adherence of SI and quarantine, making use of previous scientific literature and scales, where available, as well as self-developed items and scales. Sample items of variables can be viewed in Table 1.

1. SI non-adherence. As per public health guidance at the time of data collection, individuals were required to self-isolate after developing COVID-19 symptoms or if a co-habitant develops COVID-19 symptoms. Respondents were first asked (in separate questions) if they or someone they lived with developed COVID-19 symptoms. If they responded yes, this opened additional questions, consisting of (1) how many times they had left their house for any reason and (2) how many times family or friends had visited. As per public health guidance, respondents were asked to report on these behaviours for a period of 7 days from the day they developed symptoms (SI), and for a period of 14 days from the day a co-habitant developed symptoms (quarantine). The total infringements from going out and having visitors were pooled to create separate total counts of SI and quarantine infringements after own symptoms and after a co-habitant’s symptoms. These variables were then recoded to create binary variables of SI and quarantine non-adherence (coded 1, if a count of 1 or above infringements) vs. adherence (coded 0, if no infringements) after own symptoms and after co-habitant symptoms.
2. Demographic factors. Demographic data were collected about gender, age, ethnicity, English as first language, religion, highest qualification obtained, employment status, key worker status, and deprivation. Item wording and categories were taken directly from the England Census Rehearsal Household Questionnaire [27] where possible. Deprivation was calculated from participants’ post code using the English indices of deprivation tool [28], which returned an index of multiple deprivation decile (one being most deprived, ten least deprived).
3. Housing factors. Housing situation was captured in terms of whether participants lived in their own home, a rented home, or a rented room in a house of multiple occupancy. Furthermore, how many people participants lived with and whether they lived with someone vulnerable to COVID-19 was captured.
4. Health factors. Vulnerability was measured by asking participants whether, as defined by the UK Government, they had a medical condition which made them more vulnerable to COVID-19. Additionally, perceived susceptibility was measured via a single item adjusted from a single item measuring perceived susceptibility to cancer [29].
5. Political factors. Participants were asked which political party they voted for in the 2019 General Election with response options for all major political parties, which was collapsed as not voting for the Government or voting for the Government due to small responses to some categories. Trust in the Government (3 items, α = 0.888) was a self-developed scale covering trust in the response to COVID-19 and trust in the scientific advice. During data collection, lockdown restrictions were relaxed by the Government. To control for this and measure any effect of restrictions, the dates of participants’ submission of response were coded as total lockdown if submitted by the final day of total lockdown on Tuesday 12 May 2020. Given that participants were asked to recall SI and quarantine behaviours over two weeks, responses between Wednesday 13 May and Tuesday 26 May were coded as overlap of total lockdown and first relaxation. Responses from Wednesday 27 May recalled behaviours that were specific to the first relaxation phase and were coded as such. Data collection ended on 31st of May ahead of an additional relaxation of lockdown rules that occurred on 1 June 2020.
6. Psychological factors. COVID-19 and SI/quarantine guidelines knowledge was measured via a nine item self-developed quiz based upon the WHO COVID-19 mythbusters web portal [30] and the UK Government’s guidance on SI and quarantine rules [7]. Participants were required to identify whether each statement was ‘true, false or don’t know’, from which a score of correct answers (0–9) was calculated. Single items for self-interest and social responsibility were modified from Oosterhoff and Palmer [31]. Using the Theory of Planned Behaviour [32] as a guide, scales were self-developed for perceived behavioural control (3 items, α = 0.671) and normative pressure from family, friends and neighbours (3 items, α = 0.833). Given that the Cronbach’s alpha score for perceived behavioural control was below the threshold of 0.7 for sufficient internal consistency [33], an item was removed and modelled separately so to distinguish between control over leaving the house after having symptoms (2 items, α = 0.816) and perceived behavioural control over responsibilities, such as work and childcare.
7. Social factors. Using the Social Ecological Model [34] as a guide, participants were asked to report if during the lockdown they were receiving financial and community support if needed. Social support was measured using the multidimensional scale of perceived social support [35], with items clarified to refer to the lockdown period. Sub-scales for support from a special person (3 items, α = 0.939), family (3 items, α = 0.937) and friends (3 items, α = 0.94) were used.

#### 2.1.3. Statistical Analysis

Mean average of infringements and extent of adherence and non-adherence for SI and quarantine, as a percentage, were calculated. For categorical explanatory variables, frequencies and percentages of categories of participants adhering and not adhering to SI and quarantine rules were calculated. For numerical explanatory variables, mean values of participants adhering and not adhering to SI and quarantine rules were calculated. These descriptive statistics are available as supplementary files. Furthermore, a binary logistic regression model was run to measure the associations between explanatory variables and SI and quarantine non-adherence when holding other factors constant, from which odds ratios for each explanatory variable with the corresponding 95% confidence interval (CI) and *p*–value were generated.

### 2.2. Qualitative Analysis

#### 2.2.1. Participants and Recruitment

As part of our study on non-adherence to social distancing rules [36], a purposeful sample was developed to include a diverse range of ages, genders, ethnicities, employment statuses, and boroughs. The study also ensured the inclusion of some participants of vulnerable status, and some who reported having COVID-19 symptoms. Those participants who accepted to be contacted for an interviewed in the online survey, where randomly selected and emailed an invitation alongside a participant information sheet and an inform consent form. The email invitation asked participants to express their preference for a phone interview or via an online video platform (Zoom or Skype), and were offered a £20 Aldi voucher for their participation. Thirty individuals were interviewed between the 5th of August and the 21st of September 2020. Those individuals who confirmed their SI or quarantine status since the pandemic started where asked a separate set of questions following a specific interview guide. Interviews were conducted by Y.E., an experienced qualitative researcher with a background in health studies and public health. Interviewees were reassured that the study aimed to understand behaviours and their experiences for adhering or not to the measures.

#### 2.2.2. Data Collection

Semi-structured interviews were used with a topic guide consisting of open-ended questions, each with several prompts. The interview guide was reviewed by key stakeholders from local Public Health, Healthwatch and NHS Northcentral London Clinical Commissioning Group with whom quantitative findings were discussed during a workshop. Feedback was incorporated into the interview guide before recruitment started. The topic guide was informed by the Social Ecological Model and the Theory of Planned Behaviour. Individual level factors included questions about knowledge of symptoms, perceived behavioural control (self-efficacy and controllability), social norms (friends, family, and neighbours), perceived threat (vulnerability, susceptibility, and severity), attitude towards norms and trust in government). Interpersonal level factors included social support (friends, family, and statutory services).

All interviews were digitally recorded and transcribed verbatim. Participants have been anonymised, given identifier codes, and age ranges. For example, ‘M04, 45+’ means that interviewee is a male, interview number 04, age range 45–49 years old. Age ranges such as ‘20 s’ means (20–24 years old).

#### 2.2.3. Data Analysis

Framework analysis [37] was used for qualitative data interpretation, following its five stages (familiarization with the data, identification of a thematic framework, indexing, charting, and mapping and interpretation of themes). SI and quarantine were analysed as two separate groups. Y.E. read all the transcripts and both Y.E. and S.H. read a selection of transcripts to identify recurring themes. Both researchers independently coded the same five transcripts that led to the development of a coding framework. Coded transcripts were compared, and codes further refined and grouped into broader categories. Categories and codes were derived both inductively based on participant’s narratives and deductively from the literature that informed the research questions, i.e., the Social Ecological Model and the Theory of Planned Behaviour. The complete dataset was then manually indexed by Y.E., then S.H. independently coded two interviews to ensure consistency. Data were arranged in a case chart with one row per participants and one category and associated codes per column, alongside illustrative quotes. During the mapping and interpretation process, the authors further reviewed patterns and associations, e.g., examining the association between the two groups (SI and quarantine) to explore the existence of common themes. Generated themes were refined across the case chart and the qualitative research questions.

## 3. Results

### 3.1. Quantitative Results

#### 3.1.1. Participants

There were a total of 701 responses to the study’s questionnaire. Of these, 20 responses came from locations other than the specified North London boroughs and so were removed from the dataset, leaving a sample of 681 respondents. Given that SI and quarantine behaviours can only be measured after a participant has either experienced COVID-19 symptoms themselves or a co-habitant had, the sample was further restricted to respondents who had experienced either one or both scenarios. Out of 681 valid respondents, 255 respondents (37%) had either experienced COVID-19 symptoms or lived with someone who had symptoms (Table 2). Out of 681 valid respondents, 209 respondents (31%) had experienced COVID-19 symptoms and 150 respondents (22%) lived with someone who had experienced symptoms. Of note is that the restricted sample was highly skewed to females, with 82.7% of respondents being female (211 vs. 42 males). This over-representation reflects the well-established trend that women are more likely to participate in surveys than men [38,39]. Additionally of note is that a minority of 13.7% of the restricted sample came from Black, Asian and Minority Ethnic (BAME) populations (35 vs. 220 White), which is disproportionate to the 40.2% of the broader London population who come from BAME groups [40]. This under-representation, often observed in clinical trials and health surveys research, has been attributed to a range of factors including language, educational attainment, socioeconomic status, and lack of culturally adapted forms of engagement in the research design [41,42].

#### 3.1.2. Non-Adherence to SI and Quarantine Rules

As reported in Table 3, of the 255 respondents who either experienced COVID-19 symptoms themselves or someone they lived with did, 62% (*n* = 158) adhered to SI and quarantine rules and 38% (*n* = 97) did not adhere. On average, after experiencing symptoms themselves or someone they lived with had symptoms, participants infringed SI and quarantine rules 1.97 times (S.D. = 4.45). Of the 209 respondents who experienced COVID-19 symptoms themselves, 75.6% (*n* = 158) adhered to SI rules and 24.4% (*n* = 51) did not adhere. On average, after experiencing symptoms themselves, participants infringed SI rules 1.02 times (S.D. = 2.9). Non-adherence was nearly exclusively associated with leaving the house whereby 23.9% reporting this behaviour but only 4.3% had visitors, during the 7 days they were required to self-isolate. Of the 150 respondents who lived with someone who experienced COVID-19 symptoms, 57.3% (*n* = 86) adhered to quarantine rules and 42.7% (*n* = 64) did not adhere. On average, after someone they lived with experienced symptoms, participants infringed quarantine rules 1.93 times (S.D. = 2.9). Again, non-adherence was nearly exclusively associated with leaving the house whereby 42% reporting leaving their house, but only 4.7% had visitors, during the 14 days they were required to quarantine. That non-adherence was 75% greater and infringements 89% greater for quarantine rules (after a co-habitant experienced symptoms) compared to SI (experiencing symptoms oneself) confirms that these are distinct behaviours, thus warranting separate models of their explanatory factors. Furthermore, greater non-adherence of quarantine rules evidences the need of understanding and addressing this behaviour, which has not previously been considered in the academic literature.

#### 3.1.3. Factors Associated with Non-Adherence of SI Rules (Own Symptoms)

A logistic regression was performed to ascertain the association between demographic, housing, health, political, psychological and social factors and the likelihood that participants intentionally did not adhere to SD rules. The logistic regression model was statistically significant, χ^2^(52) = 98.461, *p* = 0.000, explained 56% (Nagelkerke *R*^2^) of the variance in non-adherence to SI rules after having experienced symptoms, correctly classified 75.6% of cases and adequately fits the data, Hosmer-Lemeshow χ^2^(8) = 6.004, *p* = 0.647. The results of the logistic regression for significant explanatory variables are reported in Table 4 and the results with all variables are presented in Table S5. When holding other factors constant, an additional level of agreement on a 7-point Likert scale about perception of control over leaving the house decreased the odds of not adhering to SI rules by 67.2%. When holding other factors constant, an additional level of agreement on a 7-point Likert scale about perception of control over responsibilities, for which it would be necessary to leave home even after symptoms, decreased the odds of not adhering to SI rules by 30.3%.

#### 3.1.4. Factors Associated with Non-Adherence of Quarantine Rules (Co-Habitant Symptoms)

A logistic regression was performed to ascertain the association between demographic, housing, health, political, psychological and social factors and the likelihood that participants did not adhere to quarantine rules after a co-habitant developed COVID-19 symptoms. The logistic regression model was statistically significant, χ^2^(52) = 107.578, *p* = 0.000. The model explained 68.8% (Nagelkerke R^2^) of the variance in non-adherence of quarantine rules and correctly classified 57.3% of cases and adequately fits the data, Hosmer-Lemeshow χ^2^(8) = 7.802, *p* = 0.453. The results of the logistic regression for significant explanatory variables are reported in Table 5 and the results with all variables are presented in Table S6. When holding other factors constant, the odds of not adhering to quarantine rules are 95.5% lower if not getting community support if needed than if getting community support when needed or not needing community support. When holding other factors constant, an additional level of agreement on a 7-point Likert scale about perception of control over leaving the house decreased the odds of not adhering to quarantine rules by 70%. When holding other factors constant, an additional level of agreement on a 7-point Likert scale about perceived susceptibility decreased the odds of not adhering to quarantine rules by 52%. When holding other factors constant, an additional correct answer on the quiz about COVID-19 and SI/quarantine knowledge increased the odds of not adhering to quarantine rules by 206.6%.

### 3.2. Qualitative Results

Interviews were conducted with 16 individuals. Ten participants (three men, six women, and one other) reported having experienced COVID-19 symptoms for which they should self-isolate for 7 days according to the guidelines. Six participants (four men and two women) had to quarantine due to living with someone who had experienced symptoms. Participant characteristics are presented in Table 6.

Interviews lasted a mean of 37 min (range 26–52 min). Interviewees’ responses covered the period March–September 2020. Three themes were identified for SI, two themes for quarantine, and two themes were common for both behaviours (see Table 7). Interpretation of the data is reported here using relevant quotations to illustrate.

#### 3.2.1. Self-Isolation

##### Uncertainty about Symptoms Experienced

Some participants were unsure about the COVID-19 symptoms they experienced, as often they had some of the symptoms but were missing one.

*‘I was unwell for about 3 or 4 weeks. I had a bad cough, I couldn’t sleep on my side because I’ll cough too much after, I had a disturbed sleep, I had a temperature but not super high just one or two degrees above normal; had a lot of pain chest; a sore throat and it was a definite dry cough. It was strange, the whole feeling of being unwell. I had other health problems I had some upset tummy at the time also so it’s possible it was some kind of gastric flu rather than Covid […] I looked at the NHS website but I wasn’t breathless, I could always breathe’* (M01, 55+).

For others, uncertainty about symptoms experienced at the beginning of the national ‘lockdown’, coupled with the idea that they were observing social distancing measures (staying at home and only leaving for permitted reasons), was enough precaution.

*‘at the beginning of lockdown I did have like a kind of weird sore throat and coughing, all four of us in the flat had maybe like 3 weeks, but I mean otherwise yeah we didn’t, I didn’t experience any other symptoms’ […] ‘It was right at the beginning, when you couldn’t really go out anyway and again there was no other symptoms so likelihood is it wasn’t actually Covid’ […] ‘the symptoms kind of actually first showed themselves like a week before lockdown came into play so I didn’t really think anything of it, and in hindsight, possibly I should have because one of the flat mates had been in Spain’* (M03, 25+).

##### Feeling Acquired Immunity

Most of the informants who had experienced COVID-19 symptoms believed they had developed natural immunity or ‘some immunity’ for the disease if they were to have symptoms again, or in feeling low risk in terms of transmission to others. Although participants did not specify if they experienced symptoms for a second time (without a confirmatory test), it was clearly presented as case of being low risk/mild case in the future.

*‘I kind of processed that if I did get it [COVID-19 in the past], you know, I’m pretty low risk in all likelihood it’s not going to… I certainly feel like I can take more risks than other people, which is also about volunteering and things, like the people that could take more of a risk should really be out helping those that can’t take the risk at all’* (M03, 25+).

*‘I think I had it. I think I have a bit of immunity. I think I will have it mildly a second time’* (F01, 60s).

##### Lack of Tests and Trust in Government

Most participants were unable to have access to a test confirming their health status, except by one, who gained access after enrolling in a trial.

*‘The government should have funded and mandated the local authorities to get involved in test and trace. In London the local authorities are going to know who the Imams are, who the Jewish community are, where the bad housing is, where the pinch points are, and they are going to have a different approach to getting information out, and testing and tracing than Serco, and loads of people that have been hired on contract. […] We are all pretty cynical of what’s going to happen next, we’re all expecting further degrees of things to be done the absolutely wrong way’* (F02, 70s).

*‘I don’t have faith in the Government, but I do have a fair understanding of the pandemic. I would comply with self-isolation in the future, but happy to have a test showed and not have to quarantine from abroad. It has to be a better system’* (F07, 45+).

#### 3.2.2. Quarantine

##### Believing Co-Habitant’s Symptoms were Mild

People who had to quarantine with someone with mild symptoms, especially if they were young adults, were prone to believe that their co-habitant was not infected with COVID-19. This led in some instances to not adhere for the two-weeks requirement.

*‘At one point my girlfriend had symptoms but only lasted for one day. We isolated for a week. It was very mild. A friend brought us some food once’* (M04, 20s).

*‘We’ve been with a friend who fell ill, he wasn’t tested but we were ninety percent certain that it was Covid. We didn’t know whether to isolate or get tested or anything, but my girlfriend had only a headache, and I didn’t have any symptoms’* (M03, 25+).

##### Isolating from the Symptomatic Only

Quarantining in a large house with sufficient space to keep individuals and facilities separated seemed to have instilled a sense of security and protection, whereby the isolation was observed indoors (i.e., from the co-habitant), but it was less observed outdoors (e.g., going out for exercise), or prompted a shorter quarantining period.

*‘During lockdown apart from one or two-week period when my grandson developed one or two symptoms, we weren’t sure, and during that time we had a stricter separation, but apart from that, we were one household. […] I basically went out once a day sometimes maybe twice a day just for a very short walk to be outside’* (M05, 70s).

*‘My daughter became ill, and didn’t have a test. We kept her quarantined for 7 days and we quarantined for 7 days as she recovered very quickly, so I am not sure whether she had it or not but she was very ill. We have a large house and a garden, there wasn’t a problem in getting on top of each other’* (M06, 65+).

##### Common Theme: Needing Food Provisions

Participants who experienced symptoms, living alone or with family or friends who had to quarantine at the same time, expressed they had to go out to buy food if they couldn’t receive support from family or friends.

*‘My husband and I negotiated when it was safe to go out. He had to be at home longer to see if he had any symptoms, and I needed to wait until my symptom has gone away. I think I did 10 days. [..] We did have to leave, we talked about it, we didn’t do it in the 1st week, but we needed to go to the supermarket. Somebody went [her or husband]. We were very careful early in the day, which shop we could go to’* (F01, 60s).

*‘In late June, for 2 days I had symptoms, strong fever and migraine, overwhelming. I didn’t have a test, because the symptoms subsided, and was in lockdown anyway, working from home, only going to the shops with a mask. I didn’t think it was Covid until later, when I spoke to a friend of mine who reported exactly the same symptoms and he tested positive’* (M07, 35+).

Similarly, for those who had to quarantine, the need for going out for food and groceries was expressed as a necessity and a source of concern.

*‘We wouldn’t see anyone, we didn’t go out, but I think once or twice I had to go out for groceries, just I couldn’t avoid it’* (M08, 35+).

*‘Probably staying at home was more difficult because we were worried about running out of groceries and not having anyone nearby and not being able to get hold of any deliveries. That was kind of more stressful definitely’* (F05, 25+).

##### Common Theme: Not Requesting Community Help

Some participants who needed to buy essential items and did not have friends or family able to help, decided not to request help from voluntary organisations or mutual aid groups.

*‘Support from the community? No, I didn’t need it because I worked out a system with my husband, but I was aware it was there’* (F01, 60s).

*‘We know there are a few [voluntary groups] but we didn’t request or approached them. Just don’t think we were vulnerable so I think other people will make better use of it, so we wouldn’t want to, you know, make use of those resources.’* (M08, 35+).

## 4. Discussion

Survey findings suggest, overall, non-adherence to quarantine measures (self-isolating for 14 days after a co-habitant developed symptoms [42.7%]) was higher than non-adherence to SI for 7 days, after developing COVID-19 symptoms (24.4%). The key associations found in the quantitative findings have been complemented and expanded by data collected through interviews, whereby participants’ accounts provided insight into their experiences and reasons underpinning these two distinct behaviours. To our knowledge, this is the first mixed-methods study about non-adherence to SI and quarantine measures in the UK.

Findings are broadly consistent with previous research reporting on key factors influencing non-adherence of both types of protective behaviours [14,16,18], but appear to differ from the above mentioned [19] CORSAIR quantitative study, which found higher rates for non-adherence to SI. This is possibly due to different measures of SI adherence — i.e., participants first had to correctly identify common symptoms (cough, high temperature, and loss of sense of smell or taste; which only half of participants did) and then report adherence to rules in the CORSAIR study, whilst in this study individuals were asked to self-determine if they had COVID-19 symptoms and, if affirmative, adherence was measured; sampling techniques (quota and convenience sampling g, respectively). In the absence of validated scales of SI, self-reported rates are likely to display variations.

In the case of non-adherence to quarantine measures, both the survey and interviews have shown that lack of control over leaving the house and not getting community support if needed increased the likelihood of non-adherence. Qualitative data also found these behaviours to affect adherence to SI measures (a common theme). Furthermore, a relationship was observed between these two behaviours (perceived behavioural control and social support), as participants reported leaving the house to buy food and groceries and, at the same time, deciding not to request help from community organisations or mutual aid groups after considering vulnerable people needed them more. Being asymptomatic and feeling healthy may induce this perception as well as prosocial values. Yet, a lack of organised support from state agencies in planning the delivery of provisions for people in quarantine, and an over reliance instead on voluntary groups or random help from neighbours has made it difficult for some individuals to fully adhere to quarantine measures.

Counterintuitively, the survey found that the more knowledge participants had on COVID-19 symptoms and SI/quarantine guidelines, the more likely they were not to adhere to quarantine measures. A possible explanation for this is that with increased knowledge comes increased conviction from an individual in self-determining that they are not contagious, so do not adhere for the full 14 days after someone they lived with developed symptoms. This supports the notion that even when people are aware of the symptoms and guidelines, it does not necessarily mean that they perceive the benefit and need to comply with the behaviour. As Azjen et al. [43] and Kelly and Barker [44] have argued, having accurate information does not always determine a self-protective behaviour. Individuals need to perceive the required behaviour, in our case quarantining for 14 days, as beneficial, wise, and effective in controlling the spread of the virus. Arguably, there seemed to be a lack of a clear rationale explaining why quarantine was necessary in messages by Government and public health agencies. As explained by those informants who did not fully adhere, they believed symptoms of co-habitants were mild or decided to isolate from the infected person only. Furthermore, not having access to testing, as reported by interviewees, may have undermined the perceived effectiveness of this measure. In addition, perceived need, a construct associated with perceived benefit in the Theory of Planned Behaviour [45], whereby individuals weigh up the costs and benefits of their behaviour, can also explain low adherence to quarantine when costs (financial, emotional, practical) are perceived to outweigh the benefits. Clarity of information is necessary, but so is believing measures are needed, and thus communication about the benefits alongside support for performing the behaviour are paramount, especially when what is demanded from individuals, as in the case of quarantine, involves the restriction of movement, income deprivation in some cases, and separation from others for a long period of time.

Non-adherence to SI was statistically significantly associated with control over leaving the house (shopping for food, medications, and exercise) and control over responsibilities which required leaving the house (e.g., work, childcare or looking after someone vulnerable). These factors resonate with some of the reasons evidenced in previous UK studies on COVID-19 [19,46]. The qualitative data, however, could not further explore leaving the house due to responsibilities as the majority of interviewees were able to work from home or were retired, nor they experienced symptoms while looking after young children. Although there is evidence that having to work outside the home, job insecurity, and the prospect of losing income increase the likelihood of non-adherence to SI [47,48], it is also relevant to consider the factors that may influence non-adherent behaviours in those groups who did not lose their incomes as their behaviours pose a risk too in the spread of the virus. Interviewees did provide insight into the reasons for having to leave the house. Uncertainty about symptoms experienced and following social distancing measures (staying at home ‘anyway’) were the most frequently reported reasons for not self-isolating. For symptoms experienced, there were individuals with clear common COVID-19 symptoms who, after seeking more advice on the NHS website, concluded that being ‘able to breathe’ meant no infection, and therefore, disregarded full isolation. Others considered the duration of symptoms experienced and as they quickly resolved, presumed of not being infected. The latter, coupled with keeping social distancing when going out shopping, was thought as enough protection for other individuals in the community. Arguably, in the absence of a test, trace and isolate strategy, self-diagnosis was shrouded with doubt. Contextual factors such as inaccessibility of medical support (explicit guidelines [7] of not visiting hospitals, GPs or pharmacies and only to consult the NHS website if symptoms got worse), and distrust in the Government’s management of the pandemic more generally (as reported by these interviewees regarding social distancing measures [36]) and the abandonment of test and trace in March, prompted individual decision-making and risk assessment. This seems to permeate to newly developed test and trace initiatives, as shown by an interim report from Liverpool on a community COVID-19 testing pilot, which found that, for some groups, distrust in Government led people to not want to take part [49]. In addition, lack of provision of food and essential needs for those having to self-isolate, as recommended early on by WHO guidance [3], made full adherence more challenging.

The qualitative data showed that those individuals that considered they had COVID-19, even without a test, felt they have acquired immunity, believed they were protected from re-infection, and were a low risk to others. This perception may have stemmed from the controversial idea of herd immunity entertained as a strategy to control the disease by the Government’s chief scientific adviser in several media interviews [50], though later retracted as a Government policy. Herd immunity seemed to have lingered in lay accounts of the disease and provided a problematic sense of reassurance, given that immunity acquired by natural infection can vary considerably amongst individuals. According to the British Society for Immunology [51], immunity is difficult to measure and there is still a need to identify the best markers for antibodies in natural infection produced by SARS-CoV-2. It is also known that after natural infection, individuals can develop ‘sterilising immunity’ (not transmitting the virus) while others only develop immunity to the disease but can become re-infected and can transmit the virus [51].

### 4.1. Implications

A few measures were implemented by the Government after data collection for this study: community tests were made more available to the public and funding to self-isolate was announced for those on low incomes at the end of September 2020. The timing of its implementation, well after the first COVID-19 wave, undoubtedly meant that SI and quarantine guidelines, back in March 2020, needed a clearer rationale of what these measures were meant to achieve. During the first national lockdown (23 March–13 May), information from Government guidelines and the NHS webpage was not sufficiently reassuring for people who had been asked to self-interpret or self-diagnose symptoms. As in other diseases, health risk communication should not be conceptualised as a one-way process of imparting information. Instead, it is recommended that health authorities adopt a two-way information system through the NHS (online chat or over the phone) to respond to people seeking advice about their symptoms. Information also needs to emphasise the reasons for SI and quarantine, in order to increase perceived benefit of the behaviour, if measures are to be perceived as promoting health rather than simply restricting personal choice and liberties. Current NHS guidelines do not provide a rationale for self-isolation [52], although tests are more widely available. Finally, and related to the latter, the perceived need of individuals in adhering to the measures could also be tackled. The UK Government should appropriately resource local authorities to work in partnership with local communities in the provision of a coordinated support for people expected to SI and quarantine, with provisions for food and other basic needs, including hotels for those who cannot effectively isolate at home. For as long as people perceive themselves as simply worse off for having to isolate, communities will remain at risk.

### 4.2. Limitations

This is the first mixed-methods study exploring behavioural and demographic factors influencing non-adherence to SI and quarantine measures and associated experiences in North London boroughs. A number of limitations, however, are present in this study that should be considered in assessing potential transferability to other contexts. The survey asked participants to self-determine if they had experienced COVID-19 symptoms, and this may be confounded by people’s accurate recognition of the symptoms. In addition, the authors acknowledge several potential biases, including the limitation of the online survey to those who could use the internet and self-reporting which introduces the potential for recall and social desirability biases. Interviews may also be affected by recall and desirability bias in responses. Moreover, although interviewees described a range of views by virtue of their age, gender, ethnicity, and boroughs, they were comparatively less representative of the London population in terms of employability status than the survey sample. This reduces the level of experiences offered given that lower income groups or those in financial hardship are known to be disproportionally affected by the pandemic. However, having access to non-deprived groups offers valuable insight into reasons for non-adherence and this should also inform evidence as all groups can pose a risk for transmission.

## 5. Conclusions

The value of this mixed-methods study lies in the assessment of behaviours whose adherence are essential for an effective control of the virus both at its initial stages and when restrictions are being relaxed to avoid a surge of new cases. Findings identified non-adherence to quarantine measures (14 days isolation after co-habitant developed symptoms) was higher than self-isolation (7 days after individuals developed symptoms) during the first wave of COVID-19 in a sample of North London residents. Non-adherence to quarantine rules was statistically associated and reported by individuals as not accessing the community support needed, perceived lack of control over leaving the house, and lack of a perceived benefit and need to follow the rules; whilst non-adherence to SI was associated with perceived lack of control over responsibilities, lack of control over leaving the house, needing food provisions, uncertainty about symptoms experienced, lack of testing and trust in Government. In addition to identifying these factors, the study has made recommendations that could assist in helping individuals to adhere. Perceived benefit to quarantine and self-isolate should be strengthened with public health messages emphasising the effectiveness and health benefits of self-isolating measures. A two-way information system to support symptoms identification for those who are experiencing symptoms or are told to quarantine. Finally, lack of perceived need to SI and quarantine should be tackled through Government-funded, locally supported packages providing assistance at different levels (financial, food, hotel quarantine for individuals who cannot properly isolate at home, and help with practical needs) for those required to isolate or quarantine. All three recommendations based on the findings of this study during the first wave of the pandemic have yet to be implemented in UK health strategies and policies.

## Figures and Tables

**Table 1:** Sample items of research variables.

Variables	Sample Items
**Symptoms development**	
Oneself	Have you developed symptoms of coronavirus at any point (even if you believe that these symptoms were not in fact coronavirus)?
Co-habitant	Has someone you live with developed symptoms of coronavirus at any point (even if they believe that these symptoms were not in fact coronavirus)?
**Self isolation**	
Non-adherence after symptoms (SI)	After developing symptoms of coronavirus, how many times did you leave your house (going to your garden does not count as leaving your house) for any reason within 7 days of developing symptoms?
Non-adherence after co-habitant symptoms (quarantine)	After someone you live with developed symptoms of coronavirus, how many times did you have family or friends visit within 14 days of developing symptoms?
**Health factors**	
Perceived susceptibility	There is a good chance that I will get coronavirus (COVID-19)
**Political Factors**	
Trust in government	I trust the UK Government in their response to COVID-19
**Psychological factors**	
COVID-19 and SI knowledge	If someone I live with develops symptoms of coronavirus (COVID-19), but I do not, I should self-isolate for at least seven days
Social responsibility	Before I act, I think about how my actions might have a negative effect on others
Self-interest	I do what I want, regardless of what others want me to do
Control over leaving the house	If I had symptoms, I would not need to leave my home for any reason
Control over responsibilities	I have responsibilities (e.g., work, childcare) for which I would need to leave my home even if I had symptoms *
Normative pressure	My immediate family would not go out for any reason if they had coronavirus symptoms
**Social factors**	
Financial support	During lockdown, are you getting the help you need with your financial situation from UK Government schemes?
Community support	During lockdown, are you getting the help you need from local community services (e.g., your council, voluntary or charitable organisations)?
Support from a special person	During lockdown, there is a special person who is around when I am in need
Support from family	During lockdown, my family really tries to help me
Support from friends	During lockdown, I can talk about my problems with my friends

* Reversed items.

**Table 2:** Characteristics of sample.

Explanatory Variables	*n*	%	Mean	Standard Deviation	Minimum	Maximum
**Symptoms Experienced by Full Sample**						
Either own or co-habitant Symptoms						
Yes	255	37.4				
No	426	62.6				
Own symptoms						
Yes	209	30.6				
No	472	69.3				
Symptoms of co-habitants						
Yes	150	22				
No	531	78				
**Demographic factors**						
Gender						
Female	211	82.7				
Male	42	16.5				
Other	2	0.8				
Age			40.68	12.492	21	75
Ethnicity						
White	220	86.3				
Black Asian & Minority Ethnic	35	13.7				
Language						
English as first language	210	82.4				
English not as first language	45	17.6				
Religion						
No religion	163	63.9				
Christian	50	19.6				
Buddhist	4	1.6				
Hindu	1	0.4				
Jewish	23	9				
Muslim	7	2.7				
Sikh	1	0.4				
Other	6	2.4				
Highest qualification obtained						
No qualifications	3	1.2				
GCSEs or equivalent	15	5.9				
A Levels or equivalent	16	6.3				
Vocational / work-related Qualification	12	4.7				
Bachelor’s degree	79	31				
Professional qualification	32	12.5				
Master’s degree	86	32.2				
Doctoral degree	16	6.3				
Employment status						
Long-term sick or disabled	8	3.1				
Retired	9	3.5				
Working as an employee from home	94	36.9				
Self-employed or freelance from home	26	10.2				
Looking after home or family	14	5.5				
Unemployed	6	2.4				
A furloughed employee	31	12.2				
A student	9	3.5				
Working as an employee in normal place of work (not home)	38	14.9				
Self-employed or freelance in normal place of work (not home)	6	2.4				
Other	14	5.5				
Key worker status						
Not key worker	180	70.6				
Key worker	75	29.4				
Deprivation (1–10)			4.47	2.15	1	10
**Housing factors**						
Housing situation						
Live in own home	121	47.5				
Live in rented home	97	38				
Live in rented room of Multiple occupancy house	37	14.5				
Number of people living with			2.84	1.43	0	9
Living with a vulnerable person						
Living with person of vulnerable health status	34	13.3				
Not living with person of vulnerable health status	221	86.7				
**Health factors**						
Health						
Vulnerable	30	11.8				
Not vulnerable	225	88.2				
Perceived susceptibility (1–7)			5.31	1.524	1	7
**Political factors**						
2019 General election						
Voted for Government	22	8.6				
Did not vote for Government	233	91.4				
Trust in Government (1–7)			2.92	1.55	1	7
Lockdown phase						
Total lockdown	101	39.6				
Overlap of total and first relaxation	96	37.6				
First relaxation	58	22.7				
**Psychological factors**						
COVID-19 and SI/quarantine knowledge (out of 9)			7.06	1.058	3	9
Social responsibility (1–7)			6.2	1.099	1	7
Self-interest (1–7)			1.76	1.109	1	7
Control over leaving the house (1–7)			5.9	1.42	1	7
Control over responsibilities (1–7)			5.98	1.758	1	7
Normative pressure (1–7)			5.98	1.117	2	7
**Social factors**						
Financial support						
Getting financial support if needed	198	77.6				
Not getting financial support if needed	57	22.4				
Community support						
Getting community support if needed	222	87.1				
Not getting community support if needed	33	12.9				
Support from a special person (1–7)			5.69	1.761	1	7
Support from family (1–7)			5.39	1.665	1	7
Support from friends (1–7)			5.44	1.454	1	7

**Table 3:** Non-adherence of SI and quarantine rules.

	Adherence (*n*)	Adherence (%)	Non-Adherence (*n*)	Non-Adherence (%)	Infringements (Mean, SD)
Isolating after own or co-habitant symptoms	158	62	97	38	1.97 (4.45)
Self-isolating within 7 days of symptoms	158	75.6	51	24.4	1.02 (2.9)
Leaving house within 7 days of symptoms	159	76.1	50	23.9	0.92 (2.65)
Friends or family visit within 7 days of symptoms	200	95.7	9	4.3	0.1 (0.51)
Self-isolating after co-habitant symptoms for 14 days	86	57.3	64	42.7	1.93 (3.66)
Leaving house within 14 days of co-habitant symptoms	87	58	63	42	1.83 (3.54)
Friends or family visit within 14 days of co-habitant symptoms	143	95.3	7	4.7	0.11 (0.557)

**Table 4:** Results of logistic regression for significant explanatory variables, with binary outcome variable of non-adherence or adherence to SI rules (own symptoms).

Explanatory Variables	Exp (B) *	95% Wald Confidence Interval for Exp (B)		Significance
		Lower	Upper	
**Psychological factors** Control over leaving the house	0.328	0.188	0.571	0.000
Control over responsibilities	0.697	0.498	0.976	0.036

* Exponentiation of the B coefficient

**Table 5:** Results of logistic regression for significant explanatory variables, with binary outcome variable of non-adherence or adherence to quarantine rules (co-habitant symptoms).

Explanatory Variables	Exp (B)	95% Wald Confidence Interval for Exp (B)		Significance
		Lower	Upper	
**Health factors** Perceived susceptibility	0.48	0.271	0.848	0.011
**Psychological factors**				
COVID-19 and SI/quarantine knowledge	3.066	1.325	7.097	0.009
Control over leaving the house	0.3	0.122	0.737	0.009
**Social factors**				
Not getting community support if needed	0.055	0.004	0.754	0.03

**Table 6:** Participant characteristics (*n* = 16).

Demographic Factors	*n*
Gender	
Female	8
Male	7
Other	1
Age	
18–29	4
30–59	9
60+	3
Ethnicity	
White EG ^1^	10
Asian EG	2
Black EG	2
Mixed EG	1
Other EG	1
Borough	
Enfield	1
Islington	3
Camden	2
Haringey	6
Hackney	2
Barnet	2
Employment status	
Working as an employee in normal place of work PT/FT	2
Working from home	10
Furloughed employee	1
Retired	3
Health factors	
Vulnerable ^2^	4
Not vulnerable	12

^1^ EG: ethnic group. ^2^ At increased risk of severe illness from coronavirus, according to Guidance on social distancing for everyone in the UK [7].

**Table 7:** Themes identified for SI and quarantine.

Self-isolation	Uncertainty about symptoms experienced
	Feeling acquired immunity
	Lack of tests and trust in Government
Quarantine	Believing co-habitant’s symptoms were mild
	Isolating from the symptomatic only
	Common themes:
	Needing food provisions
	Not requesting community help

## Data Availability

The data presented in this study are available on request from the corresponding author. The data are not publicly available because participants were informed at the time of the survey and interviews their data would be stored securely and confidentially.

## Supplementary Materials

The following are available online at https://www.mdpi.com/article/10.3390/ijerph18137015/s1, Table S1. Percentage of participants who did not adhere to SI rules and those who did by categorical explanatory variable; Table S2. Comparison of means of continuous explanatory variables between participants who did not adhere to SI rules and those who did; Table S3. Percentage of participants who did not adhere to quarantine rules and those who did by categorical explanatory variable; Table S4. Comparison of means of continuous explanatory variables between participants who did not adhere to quarantine rules and those who did; Table S5. Results of logistic regression, with binary outcome variable of non-adherence or adherence to SI rules (own symptoms); Table S6. Results of logistic regression, with binary outcome variable of non-adherence or adherence to SI rules (co-habitant) symptoms.
