# Supplementary material for: Self-Isolation and Quarantine during the UK’s First Wave of COVID-19. A Mixed-Methods Study of Non-Adherence
Source: Int J Environ Res Public Health. 2021 Jun 30;18(13):7015. doi: 10.3390/ijerph18137015 (PMC8297259; doi:10.3390/ijerph18137015)
Supplement: Supplementary file 1 [file ijerph-18-07015-s001.zip › ijerph-1252741-supplementary.pdf]

Supplementary materials:

Table S1. Percentage of participants who did not adhere to SI rules and those who did by categorical explanatory variable.

Explanatory variables	Adherence (n)	Adherence (%)	Non-adherence (n)	Non-adherence (%)
Sample	158	62%	97	38%
**Demographic factors**				
Gender				
Female	138	78%	39	22%
Male	18	60%	12	40%
Other	2	100%	0	0%
Ethnicity				
White	139	75.5%	45	24.5%
BAME	19	76%	6	24%
Language				
English as first language	133	76.9%	40	23.1%
English not as first language	25	69.4%	11	30.6%
Religion				
No religion	95	71.4%	38	28.6%
Christian	35	76.1%	11	23.9%
Buddhist	3	100%	0	0%
Hindu	1	100%	0	0%
Jewish	14	93.3%	1	6.7%
Muslim	5	100%	0	0%
Sikh	1	100%	0	0%
Other	4	80%	1	20%
Highest qualification obtained				
No qualifications	3	100%	0	0%
GCSEs or equivalent	12	85.7%	2	14.3%
A Levels or equivalent	11	84.6%	2	15.4%
Vocational / work-related qualification	6	60%	4	40%
Bachelor's degree	49	74.2%	17	25.8%
Professional qualification	21	80.8%	5	19.2%
Master's degree	47	71.2%	19	28.8%
Doctoral degree	9	81.8%	2	18.2%
Employment status				
Long-term sick or disabled	5	71.4%	2	28.6%
Retired	7	87.5%	1	12.5%
Working as an employee from home	61	81.3%	14	18.7%
Self-employed or freelance from home	16	72.7%	6	27.3%
Looking after home or family	10	100%	0	0%
Unemployed	3	75%	1	25%
A furloughed employee	16	64%	9	36%

A student	4	50%	4	50%
Working as an employee in normal place of work (not home)	22	66.7%	11	33.3%
Self-employed or freelance in normal place of work (not home)	5	100%	0	0%
Other	9	75%	3	25%
Key worker status				
Not key worker	116	76.3%	36	23.7%
Key worker	42	73.7%	15	26.3%
**Housing factors**				
Housing situation				
Live in own home	85	85%	15	15%
Live in rented home	55	67.1%	27	32.9%
Live in rented room of multiple occupancy house	18	66.7%	9	33.3%
Living with a vulnerable person				
Living with person of vulnerable health status	21	91.3%	2	8.7%
Not living with person of vulnerable health status	137	73.7%	49	26.3%
**Health factors**				
Vulnerable health				
Vulnerable	19	73.1%	7	26.9%
Not vulnerable	139	76%	44	24%
**Political factors**				
2019 General election				
Voted for Government	15	88.2%	2	11.8%
Did not vote for Government	143	74.5%	49	25.5%
Lockdown phase				
Total lockdown	63	78.8%	17	21.3%
Overlap of total and first relaxation	55	72.4%	21	27.6%
First relaxation	40	75.5%	13	24.5%
**Social factors**				
Financial support				
Getting financial support if needed	123	76.4%	38	23.6%
Not getting financial support if needed	35	72.9%	13	27.1%
Community support				
Getting community support if needed	139	76.4%	43	23.6%
Not getting community support if needed	19	70.4%	8	29.6%

Table S2. Comparison of means of continuous explanatory variables between participants who did not adhere to SI rules and those who did.

Explanatory variables	Adherence (Mean)	Adherence (S.D.)	Non-adherence (Mean)	Non-adherence (S.D.)
**Demographic factors**				
Age	42.26	12.551	39.51	11.668
Deprivation	4.49	2.178	4.47	2.004
**Housing factor**				
Number of people living with	2.77	1.341	2.41	1.283
**Health factor**				
Perceived susceptibility	5.42	1.507	5.39	1.65
**Political factor**				
Trust in government	2.85	1.519	3.13	1.69
**Psychological factors**				
Covid-19 and SI/quarantine knowledge	7.09	1.061	6.96	1.166
Social responsibility	6.31	1.04	6	1.02
Self-interest	1.72	1.094	1.71	1.026
Control over leaving the house	6.18	1.27	4.98	1.71
Control over responsibilities	6.25	1.483	5.33	2.142
Normative pressure	6.08	1.048	5.72	1.132
**Social factors**				
Support from a special person	5.69	1.817	5.41	1.913
Support from family	5.4	1.72	5.16	1.777
Support from friends	5.5	1.513	5.35	1.423

Table S3. Percentage of participants who did not adhere to quarantine rules and those who did by categorical explanatory variable.

**Explanatory variables**	**Adherence (n)**	**Adherence (%)**	**Non-adherence (n)**	**Non-adherence (%)**
Sample	158	62%	97	38%
**Demographic Factors**				
Gender				
Female	68	54.8%	56	45.2%
Male	16	66.7%	8	33.3%
Other	2	100%	0	0%
Ethnicity				
White	76	57.1%	57	42.9%
BAME	10	58.8%	7	41.2%
Language				
English as first language	75	60.5%	49	39.5%
English not as first language	11	42.3%	15	57.7%
Religion				
No Religion	45	47.9%	49	52.1%
Christian	20	71.4%	8	28.6%
Buddhist	2	66.7%	1	33.3%
Hindu	1	100%	0	0%
Jewish	13	86.7%	2	13.3%
Muslim	3	75%	1	25%
Sikh	1	100%	0	0%
Other	1	25%	3	75%
Highest qualification obtained				
No qualifications	2	100%	0	0%
GCSEs or equivalent	9	81.8%	2	18.2%
A Levels or equivalent	4	66.7%	2	33.3%
Vocational / work-related qualification	4	66.7%	2	33.3%
Bachelor's degree	21	43.8%	27	56.3%
Professional qualification	14	77.8%	4	22.2%
Master's degree	24	51.1%	23	48.9%
Doctoral degree	8	66.7%	4	33.3%
Employment status				
Long-term sick or disabled	4	100%	0	0%
Retired	1	33.3%	2	66.7%
Working as an employee from home	38	63.3%	22	36.7%
Self-employed or freelance from home	4	22.2%	14	77.8%
Looking after home or family	4	57.1%	3	42.9%
Unemployed	2	66.7%	1	33.3%
A furloughed employee	14	70%	6	30%
A student	3	50%	3	50%

Working as an employee in normal place of work (not home)	12	63.2%	7	36.8%
Self-employed or freelance in normal place of work (not home)	1	50%	1	50%
Other	3	37.5%	5	62.5%
Key worker status				
Not key worker	59	57.8%	43	42.2%
Key worker	27	56.3%	21	43.8%
**Housing factors**				
Housing situation				
Live in own home	45	60.8%	29	39.2%
Live in rented home	25	47.2%	28	52.8%
Live in rented room of multiple occupancy house	16	69.6%	7	30.4%
Living with a vulnerable person				
Living with person of vulnerable health status	18	69.2%	8	30.8%
Not living with person of vulnerable health status	68	54.8%	56	45.2%
**Health factors**				
Vulnerable health				
Vulnerable	7	70%	3	30%
Not vulnerable	79	56.4%	61	43.6%
**Political factors**				
2019 General election				
Voted for Government	11	78.6%	3	21.4%
Did not vote for Government	75	55.1%	61	44.9%
Lockdown phase				
Total lockdown	30	49.2%	31	50.8%
Overlap of total and first relaxation	35	60.3%	23	39.7%
First relaxation	21	67.7%	10	32.3%
**Social factors**				
Financial support				
Getting financial support if needed	69	59%	48	41%
Not getting financial support if needed	17	51.5%	16	48.5%
Community support				
Getting community support if needed	75	56%	59	44%
Not getting community support if needed	11	68.8%	5	31.3%

Table S4. Comparison of means of continuous explanatory variables between participants who did not adhere to quarantine rules and those who did.

Explanatory Variables	Adherence (Mean)	Adherence (S.D.)	Non-adherence (Mean)	Non-adherence (S.D.)
**Demographic factors**				
Age	39.43	13.079	37.05	10.614
Deprivation	4.58	2.262	4.36	2.058
**Housing factor**				
Number of people living with	3.37	1.389	3.28	1.24
**Health factor**				
Perceived susceptibility	5.4	1.551	5.19	1.5
**Political factor**				
Trust in government	3.15	1.668	2.67	1.43
**Psychological factors**				
Covid-19 and SI/quarantine knowledge	6.95	1.005	7.2	1.011
Social responsibility	6.34	.876	5.98	1.397
Self-interest	1.69	1.055	1.97	1.247
Control over leaving the house	6.37	.812	5.45	1.508
Control over responsibilities	6.29	1.354	5.39	2.18
Normative pressure	6.09	1.06	5.49	1.25
**Social Factors**				
Support from a special person	6.12	1.275	6.03	1.539
Support from family	5.67	1.53	5.4	1.446
Support from friends	5.66	1.265	5.33	1.365

Table S5. Results of logistic regression, with binary outcome variable of non-adherence or adherence to SI rules (own symptoms)

**Explanatory Variables**	**Exp (B)**	**95% Wald Confidence Interval for Exp (B)**		**Sig.**
		**Lower**	**Upper**	
Constant	.000			.999
**Demographic Factors**				
Gender				.242
Female				
Male	3.459	.816	14.67	.092
Other	.000	.000		.999
Age	.999	.942	1.059	.966
Ethnicity				
White				
BAME	1.731	.325	9.213	.52
Language				
English as First Language				
English Not as First Language	2.165	.511	9.177	.295
Religion				.578
No Religion				
Christian	.000	.000		.999
Buddhist	.000	.000		1.000
Hindu	1.242	.025	61.632	.914
Jewish	.000	.000		.999
Muslim	.000	.000		1.000
Sikh	.896	.017	47.628	.957
Other	5.447	1.228	24.159	.026
Highest Qualification Obtained				.383
No Qualifications				
GCSEs or equivalent	1.496E+9	.000		.999
A Levels or equivalent	769642475	.000		.999
Vocational / Work-related Qualification	3.971E+10	.000		.999
Bachelors Degree	4.080E+9	.000		.999
Professional Qualification	1.967E+9	.000		.999
Masters Degree	4.411E+9	.000		.999
Doctoral Degree	1.803E+9	.000		.999
Employment Status				.624
Long-term sick or disabled				
Retired**	.005	.000	.732	.037
Working as an employee from home	.066	.002	2.187	.128
Self-employed or freelance from home	.036	.001	2.089	.109
Looking after home or family	.000	.000		.998
Unemployed	.006	.000	1.364	.065
A furloughed employee	.123	.004	4.339	.249
A student	.126	.002	7.368	.318

Working as an employee in my normal place of work (not home)	.154	.003	7.128	.339
Self-employed or freelance in my normal place of work (not home)	.000	.000		.999
Other	.026	.000	1.672	.086
Key Worker Status				
Not Key Worker				
Key Worker	1.255	.319	4.94	.746
Deprivation	1.103	.872	1.396	.413
**Housing Factors**				
Housing Situation				.243
Live in Own Home				
Live in Rented Home	2.155	.547	8.498	.273
Live in Rented Room of Multiple Occupancy House	.612	.093	4.038	.61
Number of People Living With	.991	.609	1.612	.971
Living With a Vulnerable Person				
Living with Person of Vulnerable Health Status				
Not Living with Person of Vulnerable Health Status	5.79	.464	72.263	.173
**Health Factors**				
Vulnerable Health				
Vulnerable				
Not Vulnerable	.579	.101	3.325	.54
Perceived Susceptibility	.839	.572	1.23	.369
**Political Factors**				
2019 General Election				
Voted for Government				
Did Not Vote for Government	10.196	.267	389.817	.212
Trust in Government	1.357	.987	1.865	.06
Lockdown Phase				.234
Total Lockdown				
Overlap of Total and First Relaxation	2.525	.733	8.7	.142
First Relaxation	3.081	.704	13.483	.135
**Psychological Factors**				
COVID-19 and Social Distancing Knowledge	.812	.493	1.337	.413
Social Responsibility	.774	.478	1.252	.297
Self-Interest	.957	.568	1.614	.87
Control over Leaving the House*	.328	.188	.571	.000
Control over Responsibilities*	.697	.498	.976	.036
Normative Pressure	1.432	.786	2.61	.241
**Social Factors**				
Financial Support				
Getting Financial Support if Needed				

Not Getting Financial Support If Needed	1.233	.249	6.11	.797
Community Support				
Getting Community Support if Needed				
Not Getting Community Support If Needed	.285	.038	2.135	.222
Support from a Special Person	1.065	.746	1.522	.728
Support from Family	.936	.545	1.605	.809
Support from Friends	1.57	.728	3.389	.25

*Significant predictors of non-adherence of SI rules (own symptoms). **Significant association found, but below threshold of 10 units per cell.

Table S6. Results of logistic regression, with binary outcome variable of non-adherence or adherence to SI rules (co-habitant) symptoms.

Explanatory Variables	Exp (B)	95% Wald Confidence Interval for Exp (B)		Sig.
		Lower	Upper	
Constant	.000			1.000
**Demographic Factors**				
Gender				.242
Female				
Male	.154	.019	1.251	.08
Other	.000	.000		.999
Age	.945	.856	1.043	.261
Ethnicity				
White				
BAME	.908	.084	9.872	.937
Language				
English as First Language				
English Not as First Language	2.708	.461	15.919	.27
Religion				.606
No Religion				
Christian	.033	.000	16.031	.279
Buddhist	.000	.000		1.000
Hindu	1.03	.041	26.097	.986
Jewish	.845	.013	54.353	.937
Muslim	.000	.000		1.000
Sikh	46.028	.651	3255.416	.078
Other	2.05	.373	11.272	.409
Highest Qualification Obtained				.256
No Qualifications				
GCSEs or equivalent	.022	.000		1.000
A Levels or equivalent	.062	.000		1.000
Vocational / Work-related Qualification	.525	.000		1.000
Bachelors Degree	.102	.000		1.000
Professional Qualification	.004	.000		1.000
Masters Degree	.05	.000		1.000
Doctoral Degree	.012	.000		1.000
Employment Status				.392
Long-term sick or disabled				
Retired	6.363E+9	.000		.999
Working as an employee from home	441697730	.000		.999
Self-employed or freelance from home	6.565E+9	.000		.999
Looking after home or family	935283619	.000		.999
Unemployed	1719732.19	.000		1.000
A furloughed employee	136667899	.000		.999
A student	346474981	.000		.999

Working as an employee in my normal place of work (not home)	137030989	.000		.999
Self-employed or freelance in my normal place of work (not home)	178410501	.000		.999
Other	537003621	.000		.999
Key Worker Status				
Not Key Worker				
Key Worker	3.449	.511	23.266	.204
Deprivation	.867	.653	1.151	.323
**Housing Factors**				
Housing Situation				.022
Live in Own Home				
Live in Rented Home	4.527	.624	32.847	.135
Live in Rented Room of Multiple Occupancy House	.195	.014	2.788	.228
Number of People Living With	1.473	.785	2.766	.228
Living With a Vulnerable Person				
Living with Person of Vulnerable Health Status				
Not Living with Person of Vulnerable Health Status	.967	.145	6.43	.972
**Health Factors**				
Vulnerable Health				
Vulnerable				
Not Vulnerable	7.752	.223	269.575	.258
Perceived Susceptibility*	.48	.271	.848	.011
**Political Factors**				
2019 General Election				
Voted for Government				
Did Not Vote for Government	31.401	.606	1628.005	.087
Trust in Government	.796	.526	1.207	.283
Lockdown Phase				.379
Total Lockdown				
Overlap of Total and First Relaxation	.34	.062	1.854	.212
First Relaxation	.347	.057	2.115	.251
**Psychological Factors**				
COVID-19 and Social Distancing Knowledge*	3.066	1.325	7.097	.009
Social Responsibility	1.046	.462	2.365	.915
Self-Interest	1.542	.698	3.406	.284
Control over Leaving the House*	.3	.122	.737	.009
Control over Responsibilities	.856	.522	1.402	.536
Normative Pressure	.639	.294	1.39	.258
**Social Factors**				
Financial Support				
Getting Financial Support if Needed				

Not Getting Financial Support If Needed	1.929	.359	10.349	.444
Community Support				
Getting Community Support if Needed				
Not Getting Community Support If Needed*	.055	.004	.754	.03
Support from a Special Person	1.298	.599	2.813	.509
Support from Family	1.916	.792	4.639	.149
Support from Friends	.426	.17	1.066	.068

*Significant predictors of non-adherence of quarantine rules.
